# Peristaltic Arterial Action

**Published:** 1874-02

**Authors:** John J. Mason

**Affiliations:** New York


*NEW YORK MEDICAL JOURNAL—December*, 1873:

Peristaltic Arterial Action; Objections. — *By John J. Mason, M.D., New York.*—The physiology of that part of the nervous system which is concerned in regulating the quantity of blood in the tissues is a branch of medical science second to no other in importance, and one which will always hold high rank as an object of special study. The fact that some physicians seem weary of the subject only proves the rule; as, in literature, culture loses none of its claims on authors because it may have become to them a wearisome word. It is from no lack of intrinsic merit, therefore, that the region of the vaso-motor nerves remains so little explored, but from a lack of exact experiments which shall lay a solid basis for further research.

At present all our most distinguished physiologists agree that narrowing of the calibre of the arterioles may be induced by stimulating their accompanying nerve-fibres, and that a widening of the calibre of these vessels may be brought about by paralysis of the same nerves. Certain cases, however, of apparent widening of the calibre of the vessels are explained by one physiologist, on the supposition that an antagonism exists between the action of the sympathetic and *cerebro-spinal nervous systems;* according to another, the vessels dilate actively; another explains by introducing reflex action; another speaks of *"l'action paralysant;"* and again we have the theory of widening by passive dilatation caused by attracted blood.

CONCLUSIONS.

*a.* The experiments which I have repeated on the action of the continuous galvanic current, upon the circulation, warrant the belief that the vaso-motor nerves are affected by the continuous electric current just as are the nerves of voluntary muscles; that the alternate acceleration and retardation of the flow of blood induced by different current directions in the vessels of the frog have their cause rather in electrotonus of the vaso-motor nerves than in modified peristaltic action of the arteries.

*b.* Experiments, in which a stream of liquid is used steadily flowing from a siphon-tube, fail to fulfill a most important condition; and that, when Nature is imitated by employing a stream from an intermittent source, the action of the arteries seems rather to retard than to accelerate its flow.

*c.* Rhythmical vermicular action of the vessels in order to be efficacious as an assistant to the circulation, must be synchronous with the contractions of the heart's ventricles. No such synchronous action has ever been seen, and its existence may be justly denied, on account of the comparative slowness with which the unstriped fibres enter into contraction.

*d.* Some of the experiments on erectile structures, which MM. Onimus and Legros have published, seem opposed to their theory, while the others tend rather to prove our ignorance of the precise mechanism of erection, than to establish an active motality of the arteries as its cause.

*e.* Our present knowledge of the functions of the vasal nerves and arterioles seems capable of expression, as follows:

1. Tonicity by normal innervation (direct or reflex).
2. Narrowing of calibre by increased innervation (direct or reflex).
3. Widening of calibre by diminished or suspended innervation.
4. Widening of calibre in special cases by a mechanism unknown, but seemingly analogous to that of the depressor nerve of MM. Ludwig and Cyon.

